# Methodological Challenges in Online Trials

**DOI:** 10.2196/jmir.1052

**Published:** 2009-04-03

**Authors:** Elizabeth Murray, Zarnie Khadjesari, Ian R White, Eleftheria Kalaitzaki, Christine Godfrey, Jim McCambridge, Simon G Thompson, Paul Wallace

**Affiliations:** ^5^London School of Hygiene and Tropical MedicineLondonUK; ^4^Department of Health SciencesUniversity of YorkYorkUK; ^3^Medical Research Council General Practice Research FrameworkLondonUK; ^2^Medical Research Council Biostatistics UnitCambridgeUK; ^1^E-health UnitUniversity College LondonArchway CampusHighgate HillLondonUK

**Keywords:** Internet, randomized controlled trial, research design, alcohol drinking

## Abstract

Health care and health care services are increasingly being delivered over the Internet. There is a strong argument that interventions delivered online should also be evaluated online to maximize the trial’s external validity. Conducting a trial online can help reduce research costs and improve some aspects of internal validity. To date, there are relatively few trials of health interventions that have been conducted entirely online. In this paper we describe the major methodological issues that arise in trials (recruitment, randomization, fidelity of the intervention, retention, and data quality), consider how the online context affects these issues, and use our experience of one online trial evaluating an intervention to help hazardous drinkers drink less (DownYourDrink) to illustrate potential solutions. Further work is needed to develop online trial methodology.

## Introduction

The Internet is widely used for health-related services [1-5]. These range from simple health information sites to complex self-management programs incorporating interactive components such as decision support, social support, behavior change support [6-8], and computerized cognitive behavioral therapy [9,10]. Advantages of delivering health care over the Internet include convenience (can be used at any time of day or night), anonymity (valued by people with stigmatized conditions), and low cost.

To date, much of the research into such Internet interventions [11] has used conventional face-to-face methods of patient recruitment, randomization, and outcome assessment [12]. However, there are grounds for exploring methods of evaluation that rely totally on the Internet [13]. For example, if one of the potential advantages of an Internet intervention is that users can self-refer to it, without going through a health professional, it should also be possible for users to participate in the evaluation without going through an intermediary, thus enhancing external validity [14].

One example is the evaluation of an online intervention to help hazardous drinkers drink less (DownYourDrink, DYD) [15,16]. Data from an early cohort study of the intervention had made it clear that users appreciated the anonymity and convenience of the online environment [17]. This provided a strong rationale for ensuring that the evaluation of this intervention was also done online. In preparation for a phase 3 randomized controlled trial of DYD (DYD-RCT) [18], we undertook a phase 2 pilot trial to optimize the trial parameters [19,20]. During the pilot we undertook a number of substudies to provide empirical data to inform the final trial protocol. It was our experience that the change in trial environment, or context, from a traditional face-to-face environment to an entirely online environment had considerable impact on aspects such as recruitment, randomization, fidelity of the intervention, retention, and data quality. We also experienced two problems that are unique to undertaking research online: spamming and cybersquatting.

In this paper we use our experience from this pilot work (Textbox 1) to explore the methodological challenges that may arise in online trials of online interventions with a view to informing future research. First we address the main issues that arise in all trials (recruitment, randomization, fidelity of the intervention, retention, and data quality) and describe how the change in context impacts these, and then we touch briefly on the challenges unique to online research (Table 1).

**Case study: piloting the DYD-RCT**

**Background**

In preparation for a phase 3 randomized controlled trial of an online intervention to help hazardous drinkers drink less (DownYourDrink, DYD), we undertook a pilot phase 2 trial. The aim of the pilot was to optimize the trial parameters of recruitment, randomization, retention, and data quality. As part of our optimization procedures, we undertook various substudies within the pilot.

**Methods**

Design: Two-armed randomized controlled trial with substudies. Ethical approval was obtained from the University College London ethics committee.

Setting: The World Wide Web

Participants: Internet surfers aged 18 or over who found DYD on the Web.

Intervention: Theoretically informed interactive website aimed at helping hazardous drinkers reduce their alcohol consumption. The website contained three phases: Phase 1 applied the principles of motivational interviewing to help users reach a high-quality decision about whether and how to change their drinking. Phase 2 used behavioral self-control and computerized cognitive behavioral therapy techniques to enable the user to make the planned change, while phase 3 focused on relapse prevention. For the duration of the pilot and subsequent phase 3 trial, both intervention and comparator sites were only available to people who consented to participate in a trial.

Comparator: Text-based website containing high-quality information on the harms associated with excess alcohol consumption, but with minimal interactivity and no theoretically informed components.

Procedures: Recruitment, consent, baseline data collection, randomization, and follow-up were all undertaken entirely online using a secure site, with no offline contact between the research team and participants.

Optimization of trial parameters:

1. Recruitment
  - Focus groups of users provided feedback on the trial Web pages, including advertising, participant information, consent, and data collection.
2. Randomization and identity verification
  - User feedback on the high value users ascribed to the anonymity of the DYD site.
  - Trial of requesting voluntary provision of offline contact details (address and/or phone number): less than one-third of participants provided such details.
  - Monitoring IP addresses to look for evidence of re-registration.
  - Requiring email address validation.
3. Retention
  - Three email requests for data.
  - Emailed newsletters to participants: appeals to altruism, encouraging participants to feel part of an important endeavour.
  - Trial of offline follow-up of nonresponders (up to three letters and/or two phone calls to those who provided contact details).
  - Trial of financial incentives.
4. Data quality
  - User feedback on design of questionnaires.
  - Close collaboration between Web designers and statisticians.
  - Use of radio buttons and drop-down text, minimized use of free text, and inability to proceed until all mandatory questions answered.

**Table 1:** Summary of challenges that may arise in online trials and their possible solutions

Challenge	Possible Solutions
**Recruitment**	
Online recruitment can allow rapid recruitment of large numbers of participants, but some researchers have had difficulty in achieving target sample size.	Develop a recruitment strategy, and pilot it to determine likely recruitment rates. Achieve high visibility (eg, by including link to trial on highly visited and well-trusted websites—the host organization should have records of visitor numbers). Use mixed recruitment methods, including email, targeted online advertisements, lurking in discussion groups, and offline recruitment.
**Randomization**	
It is easy to ensure that researchers cannot subvert the randomization process and that randomization occurs after collection of baseline data; it is more difficult to ensure that participants do not register more than once, using multiple identities.	Consider the issue. Avoid building in incentives to re-register (such as advertising financial incentives for participation). Consider undertaking identity verification procedures, such as email address verification or verification of offline contact details. Monitor potential re-registration.
**Fidelity of the Intervention**	
Although the researcher has very tight control over what goes into the intervention, the user has a great deal of freedom to determine how he or she uses the intervention and, hence, to determine both “how much” of the intervention (dose) is received as well as which bits (active components) are used.	Develop a clear theoretical pathway of how the intervention is likely to work, and ensure primary and secondary outcome measures reflect this proposed pathway of action. Pilot use of the intervention to determine the relationship between how the researchers plan users to use it and how they actually use it. Monitor use of the intervention (number of log-ins, pages visited) during the trial.
**Retention**	
This is a major challenge for online trials, with very low retention rates (10%-25%) reported.	Some researchers have improved retention rates by using offline (letter or telephone) follow-up or financial incentives. These solutions all have resource implications that need considering before applying for funding.
**Data Quality**	
There are three issues to consider here: validity; alteration of psychometric properties by change of mode from paper-and-pencil to online administration; and item nonresponse.	Researchers need to determine the impact of change of mode to online administration on well-established outcome measures as part of preparation for a trial. Item nonresponse can be avoided by preventing participants from moving on to the next page until all questions are answered.
**Spamming**	
	Ensure requests for follow-up data include instructions on how to withdraw from the trial if desired.
**Cybersquatting**	
	Buy related domain names prior to starting a program of research.

## Methodological Challenges

### Recruitment

#### Conventional Trials

Recruitment is often a major challenge in conventional trials, with a recent review finding that one third of trials failed to reach the desired sample size [21]. Although there are few empirical studies of different strategies for improving recruitment [22], there are factors that are recognized as likely to enhance recruitment, including asking a clinically important question at a timely point, embedding trials in existing clinical practices, generating results that are likely to impact future practice, meeting patient needs, and having excellent organizational and communication structures [21]. A perception of equipoise among potential participants may also be important [23], as may altruism and a desire to “give something back” [24], particularly where health care is free at the point of delivery as it is in the United Kingdom.

#### Online Trials

Experience of recruitment online is varied. Recruitment to “one-off” surveys appears relatively straightforward [25], but participation in a trial requires greater commitment, both in terms of using the proffered intervention and in completing follow-up questionnaires. While some researchers report good recruitment and follow-up [26,27], others do not [28,29]. Additional problems with online recruitment include a potentially unrepresentative sample [30]. Online recruitment methods have included email invitations [28], online advertising with banner advertisements [31], invitations posted in discussion forums or user groups [28], “lurking” in discussion groups [31], and advertising on websites that specifically list trials currently looking for participants (such as ClinicalTrials.gov) [12,32]. Other researchers have opted to use more traditional offline recruitment methods [29].

#### The DYD Experience

The DYD experience was that online recruitment was very successful. In the pilot phase (8 months), there were just over 50,000 unique visitors to the DYD home page. Of these, 3734 completed all the stages leading to study entry (consent, provision of demographic data, email validation, completion of baseline outcome measures, and randomization).

The factors that we believe contributed to this good recruitment included exclusivity, user-centered design, findability, and media awareness:

1. *Exclusivity*: DYD was not freely available during the pilot and main trial; people who wished to use it were informed that it was only available as part of a research study.
2. *User-centered design*: The trial recruitment pages were developed in close collaboration with a user group, who provided detailed feedback on initial drafts of participant information and consent pages. The main message from the user group was to keep these pages brief and provide hyperlinks for those who wanted to know more about the research team, privacy policy, and other sensitive issues.
3. *Findability*: The DYD front page, with its invitation to take part in the DYD-RCT, could be reached from the home page of Alcohol Concern, the premier charity in the United Kingdom for people concerned about their alcohol consumption. There were also links to DYD from several other well-respected sites, such as those of the British Broadcasting Corporation (BBC) and UK National Health Service (NHS). We had no control over these links, which tended to come and go according to the priorities of the host organization. DYD was usually the first page of any Google search for help with alcohol problems, although again, this varied from month to month.
4. *Media awareness*: Excessive alcohol consumption and the potential hazards of drinking too much were near the top of the public health agenda during the study period. There were a series of major media stories about the harm of alcohol, with lead stories in major newspapers and the BBC. Many of these stories provided information about Alcohol Concern and/or DYD.

#### Implications for Other Researchers

The main implication for researchers planning an online trial is that a well-planned recruitment strategy is needed. Piloting can establish the likely number of visitors to a site and what proportion of visitors convert into participants. Advertising the study on the home page of a well-known and trusted charity can help ensure large numbers of visitors, and charitable endorsement is likely to have a positive impact on trust and, hence, the conversion rate of visitors to participants. Having links from numerous respected and well-visited sites is likely to be beneficial. We found it essential to have a user group to critique the trial recruitment materials. It is important to strike a balance between making the recruitment procedures easy for the participants (to enhance recruitment) and placing sufficient hurdles to ensure the participants are fully aware of what they are agreeing to and will not be surprised by subsequent requests for follow-up data (to enhance retention).

### Randomization

#### Conventional Trials

Effective randomization is the defining feature of a randomized controlled trial, with concealment of allocation being a significant component of most quality assessment measures for trials [33]. If the randomization procedure can be subverted in some way, the entire trial is jeopardized. Concealment of allocation has received a great deal of attention, with acceptable and unacceptable methods clearly defined [34].

#### Online Trials

Online trials have some advantages over conventional trials; for example, there is no way for the researchers to subvert a randomization process that is fully automated and based on computer-generated random numbers. Equally, it is easy to ensure that randomization occurs after collection of baseline data. However, online trials do have a unique problem, namely, the relative ease with which a potential participant can re-register using different identities, either to obtain access to all arms of the trial, or, if incentives for participation are on offer, to obtain multiple incentive payments. If a significant proportion of participants were to adopt this strategy, it would fatally undermine the entire trial. That this is a real, rather than hypothetical, challenge was demonstrated in a Web-based survey in which 11% of total responses were repeat submissions from existing participants. One respondent generated no fewer than 65 submissions [35]. This is part of a larger issue of identity verification—trials that are conducted entirely online have no way of independently verifying participants’ identity. Some researchers have avoided this difficulty by requiring participants to sign and return a paper consent form, sent to the participant’s home address [32].

#### The DYD Experience

User feedback from the earlier, cohort study had made it clear that DYD users valued the anonymity of the intervention [17]. We were concerned that inserting an offline consent procedure would have two negative impacts on our trial: first, it could result in the trial recruiting a population that differed systematically from our target population for whom anonymity was an important feature, and, second, sending a consent form through the post and awaiting its return would have introduced a significant time delay to the recruitment procedures, which we considered would have a negative impact on recruitment overall.

As we could not undertake an offline identify verification process, we introduced a number of processes aimed at minimizing participant re-registration:

1. We included an email validation step, to prevent people re-registering with the same email address or registering people other than themselves. However, as many people have multiple email addresses, and obtaining new email addresses is straightforward, we adopted a variety of additional strategies.
2. We tried to remove any incentive to re-register. The participant information stressed that the information provided in the two arms of the trial was the same, and it was only the format that differed. We tried to make the comparator site highly credible, with the same look and feel as the active site but with none of the psychologically enhanced interactive tools that we hypothesized were the active ingredients. We appealed to user altruism by explaining that the results of the trial would be used to inform policy and service provision within the NHS. There were no financial incentives offered at the recruitment stage for participation.
3. We attempted to monitor potential re-registrations. We did this firstly by requesting voluntary provision of offline contact details, such as address and phone number. Only one third of our participants provided either an address or a phone number, and in our subsequent substudy of offline follow-up, described below, it transpired that not all the information provided was valid. We also monitored potential re-registrations by looking at IP (Internet Protocol) addresses of users. Each IP address is unique and acts to allow electronic devices to locate and communicate with each other on an electronic network. Some computers have fixed (static) IP addresses (the computer keeps the same IP address for all time), but dynamic IP addresses (each computer is given a new IP address by the network each time it is switched on and connects to the network) are increasingly common. Moreover, re-registrations from the same IP address could be legitimate, for example, two people using the same computer, either if they cohabit or are using a publicly accessible computer. Despite these caveats, we considered that comparing the proportion of multiple registrations from the same IP address prior to the start of the trial (when there was open access to DYD) and during the trial period would give us some indication of whether re-registration was a significant problem. Before the pilot, 97% (2521/2597) of IP addresses used for registration were used to register one user only. During the pilot, this figure increased to 99% (3357/3396) of IP addresses. However, about 50% of users had a different IP address on their second log-in, reflecting use of different computers or dynamic IP addresses. These findings suggested firstly that re-registration was not increased by randomization, and secondly that re-registration amounted to no more than a few percent of registrations.

#### Implications for Other Researchers

This is an area that clearly needs considerable further work. In our experience, neither requesting optional provision of offline contact details, nor monitoring IP addresses satisfactorily addressed the issue. Equally, neither exercise provided data to suggest that this was a significant problem in reality, as well as in theory. Researchers might choose to require online participants to provide offline contact details and then use these details to contact each participant and check their identity. This approach has significant disadvantages, including deterring participants who value the anonymity of the Internet—a real issue in many areas, including alcohol consumption, drug use, sexual health, and mental health.

### Fidelity of the Intervention

#### Conventional Trials

Fidelity of the intervention is an important issue in trials of complex interventions, initially defined as interventions that consist of a number of components that may act independently or interdependently [19]. More recent thinking on what makes an intervention complex includes the number of interacting components within the experimental and control interventions, the number and difficulty of behaviors required by those delivering or receiving the intervention, the number of groups or organizational levels targeted by the intervention, the number and variability of outcomes, and the permitted degree of flexibility or tailoring of the intervention [36]. An important component of evaluations of complex interventions is a proposed mechanism of action, which predicts how, and why, the intervention works. Outcome measures can then be selected to measure change in the proposed intermediate outcomes along the pathway of action, as well as the final outcomes. Adequate interpretation of the trial findings also requires a detailed description of the intervention [20].

An additional issue is the proportion of participants who actually receive the intervention under trial. Bias is avoided by an “intention to treat” analysis, where all participants’ results are analyzed according to the treatment to which they were assigned [37], but if a substantial proportion do not receive the intervention, then power is lost and the true effect of the intervention is underestimated [38].

#### Online Trials

Internet interventions are complex interventions. One way that an Internet intervention may differ from an offline intervention is that the researcher (or intervention developer) has absolute control over what goes into the intervention. In contrast, a researcher evaluating an intervention delivered by multiple different therapists cannot be certain that each therapist is delivering the same intervention. However, with an Internet intervention, the user has a great deal of freedom in how they use the intervention, in terms of number, frequency, and duration of visits; pages used; and active participation in online interactive tools. Non-use of an intervention is a noted feature of online evaluations (the Law of Attrition [39]). Again, this differs from a therapist-delivered intervention, where the number and duration of sessions is usually standardized.

For these reasons, it is particularly important that trials of online interventions include a clear proposed mechanism of action, preferably underpinned by relevant theoretical approaches. A full description of the intervention should be provided, including any theoretical basis to its development [36]. Use of the intervention by trial participants must be carefully monitored, allowing determination of whether exposure to certain parts of the intervention is associated with change in specific intermediate outcomes.

#### The DYD Experience

The DYD intervention was based on theoretical and empirical data on effective face-to-face interventions for people at risk from their alcohol consumption [40]. A detailed description of the development of the intervention, and its format for use in the trial, has been published [16]. Automatic monitoring of each participant’s use of the intervention has been undertaken.

#### Implications for Other Researchers

As with all complex intervention trials, online trials require considerable preparatory work, including gaining a clear theoretical understanding of how, and why, the proposed intervention is likely to work [20]. This allows the researchers to identify appropriate primary outcomes and also secondary or intermediate measures.

### Retention

#### Conventional Trials

Retention in a trial, or the proportion of participants who provide follow-up data, is an important safeguard against bias. The lower the follow-up rate, the greater the risk of bias and imprecision of the estimated effect of the intervention. There is enhanced potential for bias where there are differential follow-up rates between the intervention and comparator groups.

#### Online Trials

High drop-out rates are another noted feature of online evaluations (the Law of Attrition [39]), with follow-up rates being often markedly lower in online trials than in conventional trials [30,31,41,42]. The Bull et al trial involving an online sample for an HIV prevention intervention targeting men who have sex with men reported a 15% follow-up rate at 3 months [31], while Verheijden et al had an 11% follow-up rate at 3 months in their study of a Web-based health promotion program [41]. Both these studies used email reminders only for follow-up. Studies that have used mixed methods, including postal or telephone reminders, have achieved higher follow-up rates. Glasgow et al found that a postal reminder combined with a cash incentive (US$10) more than doubled 12-month follow-up rates from 22% for email reminder only to 48% in a trial of an online weight loss program [30]. Similarly, when Couper et al in their trial of an online weight management program had only a 15% retention rate, they were able to boost follow-up among a subsample of nonresponders to 59% with telephone follow-up and to 55% with postal follow-up [42].

#### The DYD Experience

Like Bull et al, our study involved stigmatized behavior and, as described above, a population that valued their anonymity. Hence, our primary method of follow-up was by email. Participants were sent an email containing a link to follow-up questionnaires at 1 and 3 months. Nonresponders were sent up to two further email reminders at 7-day intervals with links to the full battery of outcome measures, and a final (4th) email requesting completion of the primary outcome measure only. In order to determine whether our response rate could be boosted by using additional postal or telephone reminders, we studied a subsample of 499 nonresponders at 3 months (defined as not having provided a response 40 days after the first request). Of these, 146 (29%) had provided an address, phone number, or both. Twenty-eight of these were excluded as the address or phone number proved false or incomplete (n = 8), or they responded after having been identified as nonresponders (n = 10). A further 10 were excluded as the address or phone number was non-UK based. Of the remaining 118, 17 had provided a phone number only, 22 an address only, and 79 had provided both phone number and address. Up to two postal reminders were sent to those providing an address, with an additional phone call to those providing an address and a phone number. Participants who had only provided a phone number were contacted by phone. This extensive additional follow-up yielded a total of 15 additional responses (15/499, 3%). We concluded that this was not a good use of researcher time in the context of our study.

#### Implications for Other Researchers

Poor follow-up rates are a significant challenge to online trials, particularly where all follow-up is done online. Studies of online weight loss programs have successfully boosted follow-up rates by using postal and telephone reminders for participants who did not respond to email reminders. This was not our experience with DYD, possibly reflecting the stigmatized nature of excessive alcohol consumption and our participants’ desire for anonymity, as well as our recruitment model. This issue clearly requires careful consideration, as a clear threat to valid inference in online trials. Offline follow-up is considerably more expensive and time consuming than online follow-up, so researchers planning to use mixed methods should budget accordingly.

### Data Quality

#### Conventional Trials

Researchers traditionally have two concerns about data quality. One is the validity of the data—to what extent is the information provided by participants “true”? Objective data (eg, data obtained through blood or other laboratory tests) are considered less prone to bias than self-reported, or subjective, data. However, data obtained from self-report may better reflect the intended outcome of a given intervention; for example, the effectiveness of an intervention aimed at reducing pain is best judged by patient reports of perceived pain. Using well-established, validated outcome measures enhances the external validity of a trial and can also facilitate comparing or combining data from different studies.

A second concern is the amount of missing data, in terms of item nonresponse. There has been considerable debate about how to avoid introducing bias into a study where there is missing data [43,44].

#### Online Trials

Conducting a trial entirely online has several implications for data quality. There are two implications for the validity of the data—the first is that even demographic data, such as age and gender, cannot be independently verified. The important issue here is bias, and collecting baseline data prior to randomization protects against bias in the baseline data. Systematic bias may be introduced after randomization if there is something about either the intervention or comparator that encourages differential responses to the follow-up questionnaires.

A second issue is that standard patient-completed outcome measures have usually been designed for paper-and-pencil completion. Any change in the mode of delivery of an outcome measure may change its psychometric properties [45,46].

Item nonresponse can be easily prevented in online trials by using software that does not allow participants to move on until all (mandatory) questions are answered.

#### The DYD Experience

For the reasons described above (see Randomization), we decided against offline identity verification. We focused instead on minimizing the potential for bias, by collecting baseline data prior to randomization and maximizing the credibility of the comparator intervention. Our primary outcome measure was developed specifically for online use, and we undertook a preliminary study to determine its reliability and validity [47].

All questionnaires were designed to maximize data quality by minimizing the use of free text and using drop-down menus or forced-choice options. The Web software required participants to complete all mandatory questions, and it was designed so that participants could not provide unusable data (eg, we used radio buttons, which only allowed the user to mark one answer per question). All questionnaires were piloted with a user group. At baseline, all those who entered the trial had usable data, and at follow-up, all those who completed follow-up generated data of adequate quality for analysis.

#### Implications for Other Researchers

Two collaborations were essential for high-quality data collection. The first was an active user group, who provided feedback on the draft data collection instruments. The second was the collaboration between the statisticians and the programmers, to ensure that the data collected was stored in a usable format. The great advantage of online data collection is that it obviates data entry from paper-and-pencil forms into statistical databases, thus saving a considerable amount of researcher time and money.

### Analysis

Some of the challenges inherent in online trials can best be addressed during analysis. For example, measuring levels of exposure to an Internet intervention is important for the interpretation of trial results. Since participants who never used the intervention are likely to differ systematically from those who did, they must be included in the analysis in their randomized group (the intention-to-treat principle [38]). However, there may be interest in understanding the benefit of the intervention in those who did use it. This should be explored by methods such as estimating the “complier average causal effect” (CACE), which effectively deduces the benefit of the intervention in those who did use it from the intention-to-treat results and the proportion of intervention users [48].

Analysis plans should address the potential for bias created by low follow-up rates. In the DYD trial, we have planned a series of sensitivity analyses, for example, by imputing missing outcomes, using baseline characteristics as predictors of nonresponse, and utilizing the trend in outcome across number of email reminders [18].

## Challenges Unique to Online Trials

The challenges described above demonstrate how methodological issues common to all trials are altered by the change in context from face-to-face to entirely online. In addition, we encountered two problems that were unique to online trials, namely spamming and cybersquatting.

### Spamming

Spamming is illegal in many countries, including the European Union. One software company defines an email to be spam if “(1) the recipient’s personal identity and context are irrelevant because the message is equally applicable to many other potential recipients; and (2) the recipient has not verifiably granted deliberate, explicit, and still-revocable permission for it to be sent; and (3) the transmission and reception of the message appears to the recipient to give a disproportionate benefit to the sender” [49]. Hence, for mass mailings to be legal, they should have an “unsubscribe” option easily visible. One of our participants suggested that our repeated emails requesting follow-up verged on being spam as there was no obvious way to revoke the permission to be sent emails originally granted in the consent form. As a result of this suggestion, emails requesting follow-up data were amended to include a reminder that participants could withdraw from the study at any time by following a link within the email or by sending an email to the research team (email address provided).

### Cybersquatting

Cybersquatting is “registering, trafficking in, or using a domain name with bad-faith intent to profit from the goodwill of a trademark belonging to someone else” [50]. DownYourDrink.org.uk was initially launched in September 2001, and all offline advertising ceased at the end of 2001. However, the site was increasingly accessed as its reputation grew [17]. By the end of the pilot study described here (October 2007), there were at least three cybersquatters (downyourdrink.org, downyourdrink.com, and downyourdrink.co.uk) benefiting from the DownYourDrink name. All three were sites that made money by advertising other websites. Users who visited these sites were presented with home pages that looked as if they offered appropriate alcohol services (such as information about alcohol or how to calculate units drunk), but clicking on these links took the user to a page of Web adverts. Visiting these sites also unleashed a torrent of pop-ups advertising various services. We were concerned that people who visited one of these sites while searching for the “real” DYD might think they had found the original site and be put off from further searching. We have no way of determining whether this affected a significant number of people or whether this had an adverse effect on recruitment or the reputation of DYD. However, prevention is better than cure, and our advice to other researchers would be to buy all related domain names (or at least the top-level ones like .org and .com) prior to starting a research program.

## Conclusion

Online trials are a recent development. There are strong methodological reasons for using such a design in terms of maximizing the trial’s external validity. Other benefits include easy access to large numbers of people and automated data collection, which greatly reduces the costs of the research and has the potential to improve internal validity. In our experience, the main challenges are the risks of participants subverting randomization by re-registering with multiple identities, the difficulties of collecting any objectively measured data, and the high rate of attrition, all of which challenge the internal validity of the trial. We think further methodological work addressing these challenges is needed, to enable the research community to benefit from the potential advantages of online trials.

## Abbreviations

BBC: British Broadcasting Corporation
DYD: DownYourDrink
DYD-RCT: randomized controlled trial of DYD
IP: Internet Protocol
NHS: UK National Health Service
